# Infants Born Large for Gestational Age and Developmental Attainment in Early Childhood

**DOI:** 10.1155/2018/9181497

**Published:** 2018-01-01

**Authors:** Cairina E. Frank, Kathy N. Speechley, Jennifer J. Macnab, M. Karen Campbell

**Affiliations:** ^1^Department of Epidemiology and Biostatistics, University of Western Ontario, London, ON, Canada; ^2^Children's Health Research Institute, Lawson Health Research Institute, London, ON, Canada; ^3^Department of Pediatrics, University of Western Ontario, London, ON, Canada; ^4^Department of Obstetrics and Gynecology, University of Western Ontario, London, ON, Canada

## Abstract

**Objectives:**

To investigate if an association exists between being born large for gestational age (LGA) and verbal ability or externalizing behaviour problems at ages 4-5 years.

**Method:**

A secondary analysis was conducted using the National Longitudinal Survey of Children and Youth, including singleton births in 2004-2005 followed till 4-5 years (*n* = 1685). LGA was defined as a birth weight > 90th percentile. Outcomes included poor verbal ability (scoring < 15th percentile on the Revised Peabody Picture Vocabulary Test) and externalizing behaviour problems (scoring > 90th percentile on externalizing behaviour scales). Multivariable logistic regression with longitudinal standardized funnel weights and bootstrapping estimation were used.

**Results:**

Infants born LGA were not found to be at increased risk for poor verbal ability (aOR: 1.16 [0.49,2.72] and aOR: 0.83 [0.37,1.87] for girls and boys, resp.) or externalizing behaviour problems (aOR: 1.24 [0.52,2.93] and aOR: 1.24 [0.66,2.36] for girls and boys, resp.). Social factors were found to impact developmental attainment. Maternal smoking led to an increased risk for externalizing behaviour problems (aOR: 3.33 [1.60,6.94] and aOR: 2.12 [1.09,4.13] for girls and boys, resp.).

**Conclusion:**

There is no evidence to suggest that infants born LGA are at increased risk for poor verbal ability or externalizing behaviour problems.

## 1. Introduction

Infant birth weight (BW) is both a marker of prenatal conditions and a strong predictor of neonatal health outcomes [1]. It has been noted that there is a reverse J-shaped relationship between birth weight and a number of adverse outcomes [2]. The majority of the literature has focused on those born small for gestational age (SGA). However, infants born large for gestational age (LGA) have been shown to have a higher risk of adverse obstetrical outcomes as well as future metabolic deficits [3].

There is sparse and inconclusive literature examining whether being born LGA is associated with an elevated risk of poorer developmental attainment. Using a large cohort study, Alati et al. found a positive association between being born LGA and social disorder symptoms (aOR: 1.57 [1.12, 2.20]) [4], while other studies have reported increased risk for externalizing behaviour problems (aOR: 1.39 [1.02, 2.78]) [5]. Associations have also been reported with schizophrenia [2], cognitive function [6], and autism spectrum disorder [7]. However, there have also been studies with contrasting results for each of the above associations [8–10]. Within this literature, few studies have considered pertinent confounding variables and most have focused on late childhood or early adolescence, ignoring a significant period of development [10, 11].

There are several reasons to speculate that LGA infants may be at a higher risk for poor development compared to the general population. Preexisting maternal conditions leading to LGA may themselves be related to later development. For example, maternal diabetes and obesity can result in fetal hyperinsulinemia, inflammation, and hormonal dysfunction which may play a mechanistic role within this association [12]. Also, being born LGA increases risk for childhood obesity, which has been linked to behavioural problems and poor academic achievement [5, 13]. Another hypothesized pathway arises from obstetrical and labor complications, which are more common in infants born LGA [3].

It is important to elucidate whether being born LGA can influence later development, as developmental deficiencies in childhood have been shown to have lasting consequences. Of particular interest, both externalizing behaviour problems and overall cognitive ability in early childhood have been shown to predict future academic performance [14, 15].

Accordingly, the objectives of this study were to investigate whether there is an association between being born LGA and (1) verbal ability, measured by the Revised Peabody Picture Vocabulary Test (PPVT-R) at ages of 4-5 years and (2) externalizing behaviour problems at ages of 4-5 years.

## 2. Materials and Methods

A secondary analysis was conducted using the National Longitudinal Survey of Children and Youth (NLSCY). The Social Sciences and Humanities Research Council granted access to the Statistics Canada Research Data Centre to conduct this study. Research ethics board approval was not required.

The NLSCY is a biennial survey which ran from 1994 (cycle 1) to 2008/2009 (cycle 8). The target population was children, aged 0–11 years at the time of selection, living in Canada, excluding residents of Yukon, Nunavut, Northwest Territories, Indian Reserves, or Crown Lands and children whose parents are members of the Canadian Armed Forces. The NLSCY followed the development of children from birth to early adulthood, collecting data on their health and healthcare utilization, social environment, and parents or guardians. Further details regarding the NLSCY can be found elsewhere [16].

This study used data from cycles 6–8 of the Early Childhood Development (ECD) cohort. This was a new sample of children aged 0-1 years that was added at each cycle and followed for at least three consecutive cycles examining development in early life. Any child who entered the survey in cycle 6 at ages of 0-1 years and remained in cycles 7 and 8 was eligible for inclusion. To increase the accuracy of pregnancy outcome reporting, the respondent, known as the person most knowledgeable (PMK), had to be the biological mother. Further, analyses excluded multiple pregnancies and children who were SGA.

Size for gestational age was estimated using sex-specific Canadian standards, classifying children as appropriate for gestational age (between the 10th and 90th percentile) or large for gestational age (>90th percentile) [17]. Infant's birth weight in kilograms was reported directly by mothers, while gestational age was derived from the mother's report of how many days before or after her due date she gave birth.

Verbal ability was assessed using the Revised Peabody Picture Vocabulary Test (PPVT-R), which was administered to NLSCY subjects through an in-person assessment [15]. The PPVT-R has strong psychometric properties and has also been shown to correlate well with the Wechsler Intelligence Scale for Children, Third Edition (WISC-III) (*r* = 0.76 for verbal IQ and *r* = 0.60 for full scale IQ) [18]. Children scoring less than or equal to the 15th percentile on the age-standardized score were classified as having poor verbal ability [11].

The NLSCY administered modified versions of preexisting behavioural scales assessing hyperactivity/inattention, conduct disorder/physical aggression, and indirect aggression. Each scale was based on PMK endorsement of a series of statements, to which the PMK responded “never or not true,” “sometimes or somewhat true,” or “often or very true” resulting in scores of zero to two on individual items. Scores were summed, with higher scores representing a higher level of these behaviours. A child was classified as having an externalizing behaviour problem if he or she scored in the top 10th percentile on any of the three scales used. The NLSCY reports Cronbach's alphas of 0.809, 0.774, and 0.632 for hyperactivity/inattention, physical aggression, and indirect aggression, respectively [16].

Additional variables were included in analyses as potential confounders of the association between LGA and development. These included prenatal factors (maternal age, parity, maternal education, marital status, income status, maternal race, maternal country of birth, maternal smoking, or alcohol use during pregnancy) and postnatal factors (breastfeeding duration, child stimulation as assessed by frequency of parent reading to child, maternal depression, and parenting practices). Maternal depression and parenting practices (positive interactions, ineffective parenting, and rational parenting) were measured by modified scales included in the NLSCY which have shown acceptable internal consistency and reliability [16]. A directed acyclic graph (DAG) was used to guide the analyses. DAGs are an important tool in identifying causal associations and can assist in analytic planning [19].

All statistical analyses were performed using SAS® 9.3, SAS Institute Inc., Cary, NC. All analyses were stratified by sex due to known differences in exposure and outcome, and the consideration that males and females experience social cues and health determinants differently [20]. Primary analyses included examination of frequency and mean distributions for each variable, followed by univariable logistic regression. Multivariable logistic regression was used to address the primary research questions. Models were built with block-wise entry of variables according to theoretical categories (prenatal and postnatal factors). Backwards elimination was performed at each step using a *p* value of <0.20 to retain variables. After all variables had been added and assessed, a final *p* value of <0.05 was used to fit the final model. Only final models are presented.

To account for the complex design of the NLSCY, which includes stratification and clustering, longitudinal standardized funnel weights and the bootstrapping method, a replicate based method of variance estimation, were used for all analyses.

## 3. Results

After applying all exclusion criteria, the final sample size was 1685 (52% males). In the sample, the prevalence of LGA was 18.5% (18.7% and 18.2% for boys and girls, resp.). The prevalence of poor verbal ability as measured by the PPVT-R was 17.7% for boys and 18.7% for girls, and the prevalence of externalizing behaviour problems was 21.1% for boys and 16.2% for girls. The characteristics of the final study sample can be seen in Table 1.

### 3.1. Verbal Ability

Table 2 presents univariable (unadjusted OR) and multivariable (adjusted OR) findings. There was no evidence to suggest that being LGA is associated with poor verbal ability (aOR: 1.16 [0.49,2.72] and aOR: 0.83 [0.37,1.87] for girls and boys, resp.). For girls, maternal race other than White (aOR: 2.91 [1.13,7.74]), low income status (aOR: 4.28 [1.90,9.61]), and a shorter duration of breastfeeding (aOR: 3.55 [1.68,7.49]) increased risk for poor verbal ability. Meanwhile, for boys, lower maternal education (aOR: 3.01 [1.52,5.96]) and low parental stimulation as measured by reading frequency (aOR: 3.06 [1.29,7.34]) increased risk.

### 3.2. Externalizing Behaviour

Table 3 presents univariable and multivariable findings for externalizing behaviour. There was no evidence to suggest that being LGA is associated with externalizing behaviour problems (aOR: 1.24 [0.52,2.93] and aOR: 1.24 [0.66,2.36] for girls and boys, resp.). For girls, maternal smoking during pregnancy (aOR: 3.33 [1.60,6.94]) and ineffective parenting (aOR: 1.24 [1.14,1.34]) were both seen to increase risk for externalizing behaviour problems. Maternal marital status of divorced, widowed, separated, or single was found to decrease risk in girls (aOR: 0.22 [0.07,0.68]). For boys, maternal smoking during pregnancy (aOR: 2.12 [1.09,4.13]), maternal depression (aOR: 1.05 [1.00,1.10]), ineffective parenting (aOR: 1.18 [1.08,1.28]), and irrational parenting (aOR: 1.15 [1.01,1.29]) were all found to increase risk.

## 4. Discussion

This study found no evidence to suggest that LGA is associated with either poor verbal ability or externalizing behaviour problems in children aged 4-5 years. This finding remained after adjustment for potential confounders. While these results are similar to select prior findings [8–10], they contradict other studies of associations on at least one of the measured dimensions of development [5–7].

Our findings may reflect that there is indeed no association between LGA and the two developmental outcomes investigated. However, it is also possible that our study failed to detect a true association for one of the following reasons. First, perhaps not enough time had passed to identify a true association. Second, an association may exist, but on other facets of development not yet examined. Third, while we controlled for potential confounders, it is possible that other confounders not available for consideration may exist and may have a suppressor effect, such as method of delivery or birth trauma, which have been shown to impact development and psychopathology [2]. Finally, we note that the definition of LGA varies among studies and it could be speculated that the studies which found positive associations had the opportunity of observing larger associations due to limiting the classification of LGA to >95th percentile [5, 7].

Since the purpose of this study was to examine the relation between being born LGA and developmental attainment, the factors included in the analyses were potential confounders of this association. The multivariable findings presented were hypothesis testing and should not be considered as robust models of poor verbal ability or externalizing behaviour problems in children. With that caveat, it is nonetheless important to reflect on the variables which remained in the final models. Consistent with other research, social risk factors were seen to increase risk for poor verbal ability in both girls (race, income status) and boys (maternal education). It is thought that social status affects development through differential access and quality of resources and disparities in behaviour and environment [20]. Less research has examined postnatal influences such as parental interaction within this context. While parenting practices did not retain a multivariable association, parental frequency of reading to child was associated with verbal ability in boys, consistent with research which states that reading to your child can promote language development and literacy skills [21].

Maternal smoking during pregnancy was found to increase risk for externalizing behaviours for both boys and girls. This is not necessarily a direct causal association, rather it is possibly explained by other smoking-related factors, such as socioeconomic status or home environment [22]. Unexpectedly, a marital status of divorced, widowed, separated, or single was found to be associated with a lower risk among girls. Most research has concluded that children living with marital disruption or in single parent homes fare worse on many facets of development [23]. In this study, many marital status categories had to be collapsed due to small cell sizes, so associations may be misestimated. The postnatal environment appeared to be more influential for males whereby numerous styles of parenting as well as maternal depressive symptoms all increased risk for externalizing behaviour for boys, while only ineffective parenting showed a moderate increase in risk for girls. This may be due to differential interactions with social environment among sexes [20].

The primary strength of this study is the use of a large database which was created specifically to examine early development of children and, due to that fact, collected information on many important dimensions. Also, important psychosocial variables were quantified with validated scales, allowing for consideration of confounders that other studies were not able to include, such as parental practices. This study also carefully considered the study design and complex sampling frame used by the NLSCY and incorporated appropriate statistical techniques. This allowed for a more conservative approach to parameter and variance estimation, which is especially important if there are results that are very close to the rejection threshold. Failure to include these statistical techniques may result in underestimating standard errors [16]. Finally, this study stratified all models by sex. Other studies which have failed to do so may be discounting important differences, as males and females are thought to experience and respond to social cues and health determinants differently [20].

As in all secondary analyses of predesigned surveys, researchers are limited to the questions and variables that are available to them, as well as the categorizations provided. While we accounted for many covariates, the potential role of other factors such as immigration status and maternal obesity could not be assessed, potentially excluding important variables within this association. Also, many variables were self-reported, including gestational age, leading to the potential for some inaccuracies. Research has also shown that respondents are more likely to either not respond or underreport negative behaviours [24]. Additionally, recall bias may have affected the accuracy and completeness of this information. However, it should be noted that maternal reports of birthing information, such as infant birth weight, have been shown to be accurate in this setting [25]. Finally, the longitudinal nature of this study required response in all three cycles resulting in some children being excluded from analyses. Differences were found between respondents and nonrespondents with the final study sample consisting of mothers with a higher level of education, higher rate of diabetes, and lower rate of smoking, while children and mothers were both older in the study sample. This may impact the generalizability of the findings.

## 5. Conclusion

Ongoing identification of risk factors for poor child development is important in the improvement of primary prevention and also to understand the underlying mechanisms driving associations. Although this study failed to find an association between being born large for gestational age and verbal ability or externalizing behaviour problems, it focuses on children at a younger age, examined LGA as opposed to a simple birth weight cut-off, and contributes a negative finding to a pool of mixed literature. Further research is needed to assess other dimensions of child development including outcomes such as internalizing behaviour, impulsivity, and psychopathology.

## Figures and Tables

**Table 1:** Baseline characteristics of boys and girls aged 0–3 years.

Characteristic	Girls	Boys	All
	(*n* = 803)	(*n* = 882)	(*n* = 1685)
	*n* (%) or mean (SD)	*n* (%) or mean (SD)	*n* (%) or mean (SD)
Exposure			
*Size for gestational age*			
Appropriate for gestational age	657 (81.8)	717 (81.3)	1374 (81.5)
Large for gestational age	146 (18.2)	165 (18.7)	311 (18.5)

Outcome			
*Verbal ability*			
Poor	150 (18.7)	156 (17.7)	N/A
Appropriate	653 (81.3)	726 (82.3)	
*Externalizing behaviour problem*			
Top 10th percentile	130 (16.2)	186 (21.1)	N/A
≤90th percentile	673 (83.8)	696 (78.9)	

Prenatal factors			
*Parity*			
1	309 (41.8)	342 (41.2)	651 (41.4)
2	244 (33.0)	316 (38.0)	560 (35.6)
≥3	187 (25.2)	173 (20.8)	360 (23.0)
*Mean maternal age at birth*	29.53 (5.29)	29.55 (5.46)	29.54 (5.38)
*Marital status*			
Married	519 (64.7)	598 (67.7)	1117 (66.3)
Living common-law	154 (19.2)	160 (18.2)	314 (18.7)
Widowed, separated, divorced, or single	130 (16.1)	124 (14.1)	254 (15.0)
*Maternal level of education*			
< secondary or secondary graduation	142 (18.8)	165 (19.4)	307 (19.2)
Some postsecondary	89 (11.9)	100 (11.8)	189 (11.8)
College, university, or other	521 (69.3)	583 (68.8)	1104 (69.0)
*Income status*			
Not low income	648 (80.7)	747 (84.7)	1395 (82.8)
Low income	155 (19.3)	135 (15.3)	290 (17.2)
*Maternal race*			
White	653 (87.2)	740 (87.3)	1393 (87.3)
Other	95 (12.8)	108 (12.7)	203 (12.7)
*Maternal country of birth*			
Canada	622 (82.8)	740 (87.4)	1362 (85.2)
Other	129 (17.2)	108 (12.6)	237 (14.8)
*Smoking during pregnancy*			
Yes	102 (12.8)	98 (11.2)	200 (12.0)
No	694 (87.2)	773 (88.8)	1467 (88.0)
*Alcohol use during pregnancy*			
Yes	124 (15.5)	147 (16.9)	271 (16.2)
No	672 (84.5)	724 (83.1)	1396 (83.8)
*Maternal diabetes*			
Yes	40 (5.00)	65 (7.4)	105 (6.2)
No	763 (95.00)	817 (92.6)	1580 (93.8)

Postnatal factors			
*Breastfeeding practices*			
Never or up to 4 weeks	112 (16.2)	128 (16.1)	240 (16.2)
5 weeks to 6 months	221 (31.9)	268 (33.9)	489 (32.9)
Greater than 6 months	360 (51.9)	396 (50.0)	755 (50.9)
*How often do you read to this child?*			
Rarely, never, or a few times a month	73 (9.1)	71 (8.1)	144 (8.5)
Once a week or a few times a week	183 (22.8)	243 (27.6)	426 (25.3)
Daily	547 (68.1)	568 (64.3)	1115 (66.2)
*Mean parenting scores*			
Positive interactions (0–20)	16.62 (2.89)	16.56 (2.25)	16.59 (2.27)
Ineffective parenting (0–28)	8.60 (3.52)	8.99 (3.28)	8.80 (3.40)
Rational parenting (0–16)	3.94 (2.08)	4.22 (2.04)	4.09 (2.07)
*Mean maternal depression score (0–36)*	4.03 (4.67)	4.16 (4.95)	4.10 (4.81)

Frequencies were rounded to the nearest whole number.

**Table 2:** Unadjusted and adjusted odds ratios (95% CI) for poor verbal ability in girls and boys aged 4-5 years.

Variables	Girls		Boys	
	Unadjusted ORs (95% CI)	Adjusted ORs (95% CI)	Unadjusted ORs (95% CI)	Adjusted ORs (95% CI)
		*R* ^2^ = 0.1342		*R* ^2^ = 0.0539
		Adjusted *R* ^2^ = 0.2224		Adjusted *R* ^2^ = 0.0926
*Size for gestational age*				
Appropriate for gestational age	Ref	Ref	Ref	Ref
Large for gestational age	1.08 (0.43, 2.69)	1.16 (0.49, 2.72)	0.85 (0.39, 1.82)	0.83 (0.37, 1.87)

Prenatal				
*Marital status*				
Married	Ref	NS	Ref	NS
Living common law	1.36 (0.71, 2.58)		1.81 (0.99, 3.30)^a^	
Widowed/separated/divorced/single	4.87 (1.88, 12.66)^b^		2.44 (0.79, 7.59)^a^	
*Maternal country of birth*				
Canada	Ref	NS	NS	NS
Other	2.01 (0.86, 4.68)^a^			
*Maternal race*				
White	Ref	Ref	NS	NS
Other	4.19 (1.69, 10.39)^b^	2.91 (1.13, 7.74)^b^		
*Maternal level of education*				
<Secondary or secondary grad	Ref	NS	3.10 (1.61, 5.96)^b^	3.01 (1.52, 5.96)^b^
Some postsecondary	1.60 (0.62, 4.12)		2.04 (1.05, 3.96)^b^	1.93 (0.98, 3.79)^a^
College/university/other	2.03 (0.73, 5.64)^a^		Ref	Ref
*Income status*				
Low income	3.87 (1.80, 8.32)^b^	4.28 (1.90, 9.61)^b^	2.48 (1.15, 5.36)^b^	NS
Not low income	Ref	Ref	Ref	
*Parity*				
1	Ref	NS	Ref	NS
2	1.13 (0.46, 2.78)		1.37 (0.73, 2.54)	
≥3	2.856 (1.25, 6.53)^b^		2.23 (1.08, 4.61)^b^	

Postnatal				
*Breastfeeding practices*				
Never or up to 4 weeks	1.91 (0.52, 6.94)	1.90 (0.56, 6.49)	NS	NS
5 weeks to 6 months	3.47 (1.67, 7.23)^b^	3.55 (1.68, 7.49)^b^		
Greater than 6 months	Ref	Ref		
*How often you read to this child?*				
Rarely, never, or a few times a month	2.22 (0.77, 6.45)^a^	NS	3.26 (1.42, 7.46)^b^	3.06 (1.29, 7.34)^b^
Once a week or a few times a week	2.69 (1.41, 5.17)^b^		2.82 (1.44, 5.49)^b^	1.93 (0.98, 3.81)^a^
Daily	Ref		Ref	Ref
*Maternal depression*	NS	NS	1.07 (1.01, 1.13)^b^	NS
*Parenting score (rationality)*	1.12 (0.96, 1.31)^a^	NS	NS	NS

^a^ *p* < 0.2, ^b^ *p* < 0.05, and NS = not significant at *p* < 0.05 level.

**Table 3:** Unadjusted and adjusted odds ratios (95% CI) for externalizing behaviour problems in girls and boys aged 4-5 years.

Variables	Girls		Boys	
		Adjusted		Adjusted
	Unadjusted ORs (95% CI)	ORs (95% CI)	Unadjusted ORs (95% CI)	ORs (95% CI)
		*R* ^2^ = 0.0863		*R* ^2^ = 0.1024
		Adjusted *R* ^2^ = 0.1470		Adjusted *R* ^2^ = 0.1591
*Size for gestational age*				
Appropriate for gestational age	Ref	Ref	Ref	Ref
Large for gestational age	1.11 (0.49, 2.49)	1.24 (0.52, 2.93)	0.97 (0.53, 1.79)	1.24 (0.66, 2.36)

Prenatal				
*Marital status*				
Married	Ref	Ref	Ref	NS
Living common law	1.51 (0.79, 2.86)	1.61 (0.77, 3.36)	1.53 (0.84, 2.79)^a^	
Widowed/separated/divorced/single	0.39 (0.15, 1.01)^b^	0.22 (0.07, 0.68)^b^	0.81 (0.37, 1.81)	
*Maternal diabetes*				
Yes	3.27 (1.34, 7.98)^b^	NS	NS	NS
No	Ref			
*Smoking during pregnancy*				
Yes	1.92 (0.99, 3.74)^a^	3.33 (1.60, 6.94)^b^	2.33 (1.27, 4.29)^b^	2.12 (1.09, 4.13)^b^
No	Ref	Ref	Ref	Ref
*Alcohol use during pregnancy*				
Yes	1.88 (1.01, 3.48)^b^	NS	NS	NS
No	Ref			

Postnatal				
*How often you read to this child?*				
Rarely, never, or a few times a month	2.47 (0.83, 7.39)^a^	NS	1.55 (0.68, 3.53)	NS
Once a week or a few times a week	1.41 (0.78, 2.54)		1.46 (0.87, 2.47)^a^	
Daily	Ref		Ref	
*Maternal depression*	1.05 (0.99, 1.11)^a^	NS	1.06 (1.01, 1.11)^b^	1.05 (1.00, 1.10)^b^
*Parenting (positive interactions)*	0.86 (0.76, 0.98)^a^	NS	0.92 (0.82, 1.03)^a^	NS
*Parenting (ineffective)*	1.18 (1.09, 1.28)^b^	1.24 (1.14, 1.34)^b^	1.24 (1.15, 1.33)^b^	1.18 (1.08, 1.28)^b^
*Parenting (rationality)*	1.22 (1.06, 1.39)^b^	NS	1.29 (1.16, 1.43)^b^	1.15 (1.01, 1.29)^b^

^a^ *p* < 0.2, ^b^ *p* < 0.05, and NS = not significant at *p* < 0.05 level.
